# ^18^F-PEG1-Vinyl Sulfone-Labeled Red Blood Cells as Positron Emission Tomography Agent to Image Intra-Abdominal Bleeding

**DOI:** 10.3389/fmed.2021.646862

**Published:** 2021-07-05

**Authors:** Xinyi Zhang, Li Wang, Wenhui Fu, Yue Feng, Chengrun Zeng, Liu Zhou, Tao Zhang, Tingting Xu, Jianpeng Cao, Zibo Li, Yue Chen

**Affiliations:** ^1^Department of Nuclear Medicine, The Affiliated Hospital of Southwest Medical University, Luzhou, China; ^2^Nuclear Medicine and Molecular Imaging Key Laboratory of Sichuan, Luzhou, China; ^3^Academician (Expert) Workstation of Sichuan, Luzhou, China; ^4^School of Pharmacy, Southwest Medical University, Luzhou, China; ^5^Department of Radiology, Lineberger Comprehensive Cancer Center, and Biomedical Research Imaging Center, University of North Carolina, Chapel Hill, NC, United States

**Keywords:** ^18^F-vinyl sulfone, red blood cell, blood pool imaging, intra-abdominal hemorrhage, positron emission tomography (PET)

## Abstract

^18^F-Labeled blood pool agents (BPAs) have attracted great attention for identifying bleeding sites. However, many BPAs are not sufficiently evaluated partially due to the limitations of labeling methods. In our previous work, we noticed that ^18^F-PEG1-vinyl sulfone (^18^F-VS) could efficiently label red blood cells (RBCs) *ex vivo* and *in situ*. However, its application as BPA is not fully evaluated. In this study, we systematically explored the feasibility of using ^18^F-VS-labeled RBCs as a positron emission tomography (PET) BPA for intra-abdominal bleeding diagnosis. In brief, we first optimized the labeling conditions, which lead to an 80% labeling yield of RBCs after incubating with ^18^F-VS in phosphate-buffered saline (PBS) at 37°C for 20 min. ^18^F-VS-labeled RBCs were found to be stable *in vitro*, which could simplify its transportation/storage for *in vivo* applications. In normal rat PET study, the cardiovascular system could be clearly imaged up to 5 h post injection (p.i.). An intra-abdominal hemorrhage rat model demonstrated that the ^18^F-VS-labeled RBCs clearly showed the dynamic changes of extravascular radioactivity due to intra-abdominal hemorrhage. Validation in the model of gastrointestinal bleeding clearly demonstrated the great potential of using ^18^F-VS-labeled RBCs as a BPA, which could be further evaluated in future studies.

## Introduction

Blood pool imaging (BPI) is widely used in preclinical and clinical research including the detection of gastrointestinal bleeding (1), blood volume measurement (2), evaluation of cardiac function (3, 4), localization of hemangiomas (5), cerebral blood flow (6, 7), detection of infection (8), or lymphoma (9). Various kinds of BPI agents have been developed, including ^18^F-FDG-, ^111^In-oxine (8), or ^99m^Tc-HMPAO-labeled leukocytes (10); ^68^Ga-NOTA-NEB-(9) and ^111^In-oxine-labeled platelets (11); ^99m^Tc-PYP-labeled red blood cells (RBCs) (12); radionuclide-labeled peptides (13); and magnetic resonance angiography-based BPI agents (14). RBCs represent promising BPI agents due to their good stability and easy availability. In fact, radionuclide-labeled RBCs have been used to obtain functional information of the cardiovascular system through quantitative analysis of BPI. During the past few decades, radionuclide-labeled RBCs [such as ^51^Cr-RBCs (15), ^99m^Tc-PYP-RBCs (16), and ^68^Ga-oxine-RBCs (17)] have shown great progress in preclinical and clinical research applications. They have been used for cardiac function evaluation, diagnosis of hemangioma and digestive tract bleeding, cerebral blood flow measurement, and spleen imaging and positioning. Despite the progress, limitations of these BPI-agents include 1) unstable labeling yield: for example, the labeling rate of ^99m^Tc-PYP-RBCs was greatly affected by the type and dose of the drug applied (18) and 2) the resolution of single-photon emission nuclide ^99m^Tc often resulted in reduced image quality compared with the positron emission tomography (PET) nuclide (19). Therefore, researchers have been trying to develop BPI agents based on PET nuclides. ^11^C, ^13^N, and ^68^Ga have been used to label RBCs, but their short half-lives or image resolution still limited their widespread clinical application to a certain extent (20, 21).

The positron nuclide ^18^F has the advantages of good image quality and suitable half-life (109.8 min) for commercialization and transportation compared with other positron nuclides. Moreover, the resulting carbon–fluorine bonds generally have reasonable stability, which could be advantageous for BPI (22). However, there are only a few ^18^F-labeled RBCs reported as BPI agents partially due to the limitation of the labeling method (23). Therefore, there is a need to find new methods that could lead to easily prepared ^18^F-labeled BPI agents for preclinical and clinical diagnosis applications.

Previously, we established a new method for site-specific labeling of thiol groups based on ^18^F-labeled vinyl sulfone (^18^F-VS). Both peptides and proteins were found to react with ^18^F-VS through the Michael addition in an aqueous system (24). Moreover, the resulting conjugates are stable in the aqueous solution and would not be hydrolyzed in a neutral solution like maleimide conjugates (25). Recently, it was also found that ^18^F-VS could react with amino groups in addition to thiol groups even though the reaction is slower (26). Interestingly, we observed that ^18^F-VS could efficiently label RBCs *in vitro* and *in vivo*. Despite the observation, the conditions to label RBCs were not optimized, and its application as BPI was not studied.

In this study, we evaluate the use of ^18^F-((2-(2-fluoroethoxy)ethyl)sulfonyl)ethene (^18^F-PEG1-vinyl sulfone [^18^F-VS]) for RBC labeling, which are then applied as a new BPI agent for abdominal hemorrhage imaging in animal models.

## Materials and Methods

All chemicals involved in the synthesis were of reagent grade and purchased from Aladdin Bio-Chem Technology (Shanghai, China) or Sigma. The radiochemical purity was documented by high-performance liquid chromatography (LC-16). A gamma counter (CAPRAC-t, Huaruisen Technology Development Co., Ltd., Beijing, China) and a dose calibrator (CRC-15R, Capintec Inc., Florham Park, NJ) were used to measure the radioactivity of the samples. Mouse and rat data acquisitions were performed with a micro-PET/computed tomography (CT) scanner (Inveon, Siemens, Munich, Germany). Healthy Kunming mice (20 g ± 2 g) and Sprague-Dawley (SD) rats (120 g ± 12 g) were provided by the Animal Experimental Center of Southwestern Medical University (Animal License SCXK 2018-17), and all studies were approved by the Ethics Committee of Southwest Medical University. The precursor 2-(2-(vinyl sulfonyl)ethoxy)ethyl 4-nitrobenzene sulfonate was synthesized and characterized using the method described in the literature (24).

### Synthesis of Intermediate Synthon [^18^F]-((2-(2-Fluoroethoxy)Ethyl)Sulfonyl)Ethene

The [^18^F]F^−^ produced from cyclotron was trapped on a QMA cartridge and then eluted by a tetrabutyl ammonium bicarbonate (TBAB) solution in water and acetonitrile. The resulting tetrabutylammonium fluoride ([^18^F]TBAF) solution was thoroughly dried by heating with anhydrous acetonitrile with N_2_ blow three times and then redissolved in anhydrous acetonitrile. [^18^F]TBAF (150 mCi in acetonitrile) was added to the solution of 2-(2-(vinyl sulfonyl)ethoxy)ethyl 4-nitrobenzene sulfonate (5 mg) in anhydride acetonitrile (80 μl) in a cap-sealed v-vial. The reaction mixture was heated at 85°C for 15 min. After cooling down, 1.0 ml of water was added, and the reaction mixture was loaded on HPLC for purification (column: type, AQ 5 μm; size, 4.6 mm × 250 mm, col. no. A6AD 10292; solvent A: 0.1% trifluoroacetic acid water; solvent B: 0.1% trifluoroacetic acid acetonitrile; 0–2 min: isocratic elution of 15% solvent B; 2–22 min, 15–95% of solvent B; flow rate: 3 ml/min). The desired product ^18^F-VS has a retention time of 11 min, and the average radiochemical yield is 31%. The acetonitrile in the product was removed under vacuum, and the final product was reformulated with 1 × phosphate-buffered saline (PBS, pH 7.3). The radiochemical purity analysis of ^18^F-VS was performed using HPLC (Supplementary Figure 2).

### RBCs Preparation

The rats were anesthetized with isoflurane, and 4 ml of blood was collected from the heart using an injection needle. Heparin (1,000 IU/kg body weight) was used to prevent blood clotting. After centrifugation (400 g for 10 min at 20°C), RBCs were located at the bottom of the tube. The plasma was separated from RBCs and stored for subsequent post-labeling stability studies. The buffy coating, which contains most of white blood cells and platelets, was removed, leaving the RBC layer undisturbed.

### Optimization of Labeling Conditions

The separated RBCs and ^18^F-VS solution (74 MBq, 600 μl) were mixed thoroughly and then divided into eight tubes and incubated separately at 0°C and 37°C (*n* = 4 in a group). The incubation time varied from 0 to 60 min, with 10-min intervals. At the corresponding incubation timepoint, 20 μl of the corresponding suspension was removed and mixed with an additional 100 μl PBS solution. Then, the mixture was subjected to centrifugation. Radioactivity of the supernatant and that of the erythrocyte sediment were measured separately. Optimal conditions for labeling RBCs with ^18^F-VS were determined and used thereafter for further evaluation.

### Post-Labeling Stability of ^18^F-VS-RBCs

Post-labeling stability was evaluated by calculating the radioactivity released from ^18^F-VS-RBCs. The optimized labeling condition was detailed below: the centrifugal-washed RBC suspension (3 ml) and ^18^F-VS solution (74 MBq in 600 μl) were mixed thoroughly and incubated at 37°C for 30 min. Then ^18^F-VS-RBCs were washed three times with PBS (8 ml), and ^18^F-VS-RBCs was isolated. To the purified ^18^F-VS-RBCs, plasma solution was added and mixed. ^18^F-VS-RBC suspension was divided into two tubes and incubated for 0 to 180 min (30-min intervals) at 0° and 37°C, respectively. After the corresponding incubation time, ^18^F-VS-RBC suspension was cooled and uniformly resuspended. Then, the radioactivity of ^18^F-VS-RBC suspension (10 μl) was measured (*n* = 4). The remaining ^18^F-VS-RBC suspension was centrifuged at 450 g for 2 min, and the supernatant (10 μl) was sampled (*n* = 4). Radioactivity counts of the RBC suspension and supernatant were simultaneously measured. The release fraction was calculated according to the following formula: release fraction (%) = (radioactivity of the supernatant/radioactivity of initial RBC pellet) × 100%. The supernatant at 30 and 120 min timepoints were also analyzed by radio-HPLC. For the sample at the 120 min timepoint, elution from HPLC was collected per minute and counted by a gamma counter due to the low radioactivity.

### Incubation of ^19^F-VS With RBCs

^19^F-VS was prepared with the previously reported method (26), which was then added to 5 μl RBCs in 2 ml saline to form a final concentration of ^19^F-VS at 10 and 100 μM. No ^19^F-VS was added in the blank control. All RBCs in the Petri dishes were incubated at 4°C, and the shape of RBCs was observed at the 2, 6, 12, and 24 h timepoints.

### PET Imaging of Normal Rats

Normal SD rats (120 ± 12 g) were used in this study. All rats were anesthetized by inhalation of isoflurane, which was maintained throughout the imaging procedure. The imaging study was performed using a small-animal PET system. SD rats were placed on a fixed plate in the supine position for scanning. ^18^F-VS-RBCs (24.8 ± 2.6 MBq and 400 μl) was injected through the tail vein. At the same time, images were continuously acquired for 60 min using the list mode. The list mode data were reconstructed using a dynamic sequence (30 frames, 60 s). After reconstruction, regions of interest (ROIs, mm^3^) of the heart, blood vessels, and spleen were obtained using the software provided by the supplier (Inveon Research Workplace 4.2, Siemens). The values were presented as dose per gram of organ (% ID/g).

### Imaging Study of the Rat Intra-Abdominal Hemorrhage Model

A glycerin enema was injected into the colon through the anus of rats to promote defecation about 30 min before the image acquisition. Then, 22.5 MBq of tracer (^18^F-VS-RBCs with 1,000 IU/kg body weight heparin for anticoagulation) was injected through the tail vein and the PET/CT dynamic acquisition was started simultaneously. List mode data were acquired for 60 min. Under steady-state BPI conditions (10–15 min after injection), a 12-gauge lumbar puncture needle was used to manually puncture the colon wall through the anus to cause abdominal bleeding.

### Image Analysis

For the abdominal hemorrhage model, the data acquisition and reconstruction were performed using the procedures described above. The ROI was drawn in the corresponding bleeding area. Radioactivity was presented using %ID/g, which was then then used to obtain the corresponding time–activity curve. The post-bleeding image (58–60 min after injection) and pre-bleeding image (10–20 min after injection) were subtracted with the PMOD software (Zurich, Switzerland) to measure the radioactivity of the bleeding site in the abdominal hemorrhage model. Negative values of the pixels in the subtracted image were replaced with zero values. Then, the total radioactivity of the abdominal hemorrhage image was presented as the percentage of the injected dose after excluding bladder radioactivity.

### Statistical Analysis

Quantitative data were presented as mean ± standard deviation. Statistical analyses were performed using the SPSS Statistics 20.0 software package (IBM, Chicago, IL). The significance level was set to 0.05.

## Results and Discussion

### ^18^F-VS Preparation

Similar to previous reports, the ^18^F-VS was obtained through a nucleophile substitution of VS-ONs with [^18^F]TBAF. The reaction mixture was purified using HPLC (Supplementary Figure 1), and the resulting ^18^F-VS was obtained in 25–40% yield with ≥99% radiochemical purity and a retention time of 12.2 min (Supplementary Figure 2).

### Evaluation Labeling Efficiency of ^18^F-VS-RBCs

**Figure 1:**
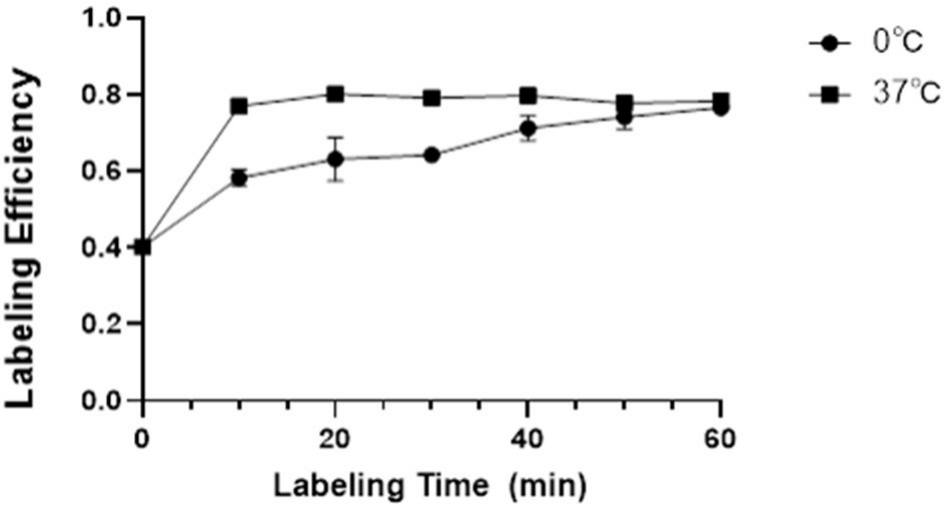
The LE and its influencing factors. Relationship between labeling temperature, incubation time, and the LE of ^18^F-VS-RBCs (*n* = 4).

As a new BPI agent, it is important to optimize the labeling conditions to maximize the yield of ^18^F-VS-RBCs. It is also important to understand the release profile from RBCs under different conditions (Figure 1).

The labeling efficiency (LE) of ^18^F-VS on RBCs was higher with an incubation temperature of 37°C compared with 0°C. Prolonging the incubation time would increase the LE steadily, which would then reach a plateau. At 37°C, the LE is 76.93% (±1.66%), 80.10% (±1.08%), and 79.11% (±1.63%) at 10, 20, and 30 min, respectively. At 0°C, the yield is 58.17% (±2.18%), 63.12% (±5.67%), and 64.23% (±1.70%) at 10, 20, and 30 min, respectively. Further increasing the incubation time led to a decreased non-decay-corrected yield. Considering the increased radioactivity decay over time, we concluded that the optimal labeling condition for ^18^F-VS-RBCs is 20 min incubation at 37°C.

### Evaluation *in vitro* Stability of ^18^F-VS-RBCs

**Figure 2:**
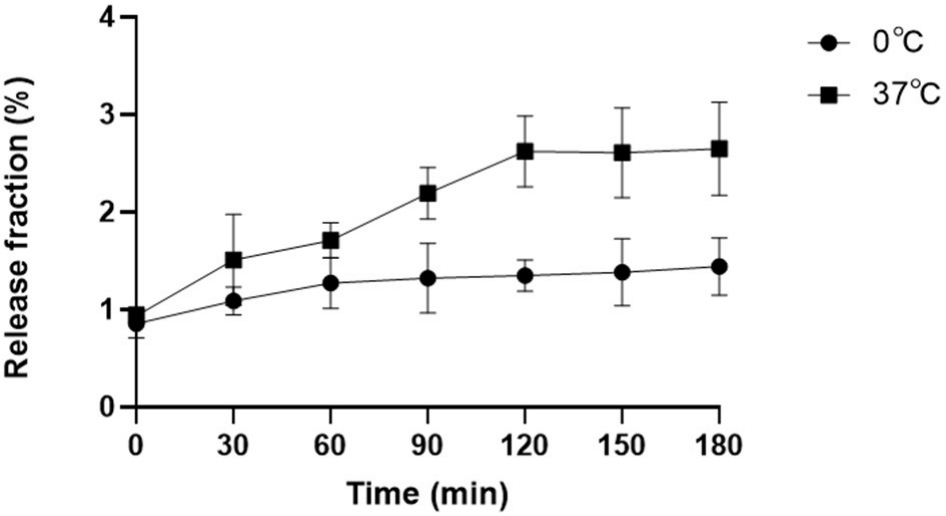
Post-labeling stability analysis. The released radioactive fraction of ^18^F-VS-RBCs when incubated in plasma at 37° or 0°C for 0–180 min (*n* = 4).

To evaluate the *in vitro* stability of ^18^F-VS-RBCs, a post-labeling stability experiment involving different storage temperatures was performed (Figure 2). In this study, the released ^18^F-containing fraction from ^18^F-VS-RBCs was evaluated for 180 min. Samples were incubated at 37° and 0°C, respectively, to simulate the temperature of the human body and the potential transportation/storage conditions using ice packs. ^18^F-VS-RBC suspension (10 μl) was then taken for analysis at different incubation timepoints.

The release of ^18^F was relatively small but noticeable. Incubation at 37°C for 0, 60, 120, and 180 min resulted in release fractions of 0.94% (±0.09%), 1.71% (±0.18%), 2.62% (±0.36%), and 2.65% (±0.48%), respectively. The corresponding samples incubated at 0°C had release fractions of 0.86% (±0.15%), 1.27% (±0.26%), 1.35% (±0.16%), and 1.44% (±0.29%), respectively. ^18^F-VS-RBCs showed better stability when stored at 0°C compared with 37°C. This indicated that ^18^F-VS-RBCs should be stored at 0°C between preparation and injection. The agent could be rewarmed before injection for *in vivo* research.

Overall, the released fractions of ^18^F-VS-RBCs in the *in vitro* study were rather low. Thus, we concluded that ^18^F-VS-RBCs were relatively stable *in vitro*. Moreover, HPLC analysis of the supernatant indicated that the released fraction only had a small amount of ^18^F-VS in addition to some unknown radioactive fractions (Supplementary Figure 3). This observation indicated that ^18^F-VS likely reacted with RBC through a covalent bond instead of passive absorption. As the released radioactive fraction may be excreted in the urine or absorbed by extravascular tissue after injection, imaging should be performed at an early timepoint if possible.

### Evaluate the Toxicity of ^19^F-VS on RBCs

To evaluate the toxicity of the tracer, the ^19^F-VS was prepared and incubated with RBCs at 4°C in saline. The shape of RBCs was observed, and images of RBCs were recorded at each timepoint (Supplementary Figure 5). As shown in Supplementary Figure 5, the shape of RBCs stayed complete, and there was no obvious broken RBCs observed, indicating that ^18^F-VS has no apparent toxicity on RBCs.

### Evaluation of ^18^F-VS-RBCs for PET Imaging

Under optimal conditions, the average time from drawing blood to intravenously injecting ^18^F-VS-RBCs was ~60 min. Microscopic examination showed that the morphology of ^18^F-VS-labeled RBCs was normal without abnormal aggregation. The final ^18^F-VS-RBC suspension (400 μl) had a radioactivity of 25.9 MBq (±3.7 MBq) and an LE of 70.09% (±0.61%) (*n* = 4).

#### PET Imaging of Normal Rats

**Figure 3:**
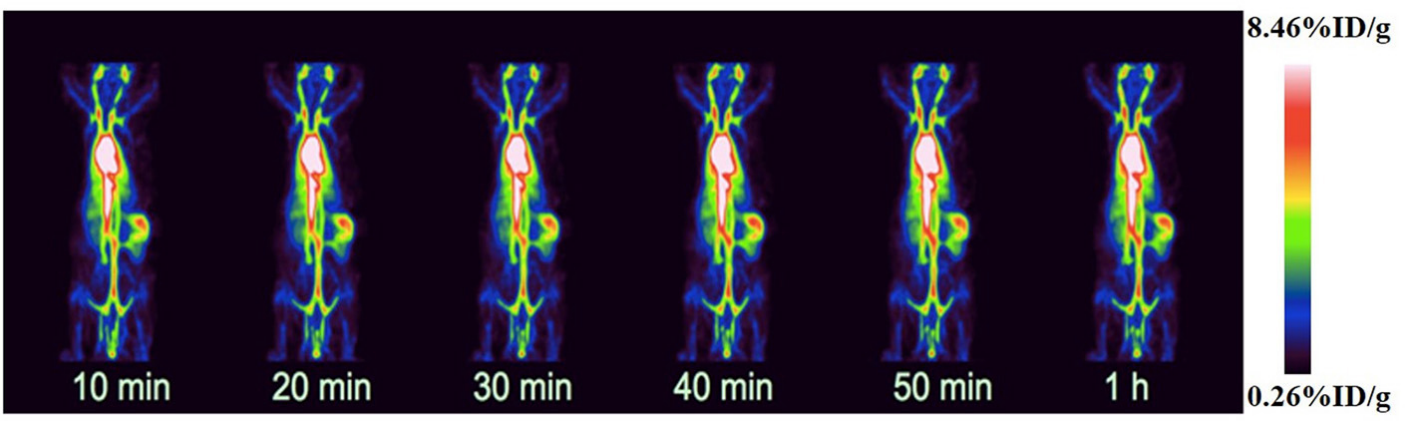
Dynamic scan of normal rats using micro-PET/CT after injection with ^18^F-VS-RBCs.

In order to evaluate the distribution of ^18^F-VS-RBCs in the cardiovascular system and the changes of radioactivity in the blood pool, we performed ^18^F-VS-RBC imaging in normal rats. The maximum-intensity projection image and biodistribution of ^18^F-VS-RBCs within 60 min after injection are shown in Figure 3 and Table 1, respectively. The cardiovascular system in normal rats had a strong uptake of ^18^F-VS-RBCs. A high uptake was obtained within 20 min, which was maintained stably at late time-points. Compared with that at 10 min, cardiac radioactivity at 60 min only decreased by ~0.7%. The radioactivity of most organs remained relatively constant within 60 min. The results indicated that ^18^F-VS-RBCs had a good stability *in vivo*. Within 60 min after tracer injection, atrium and spleen radioactivity was higher than that in the liver and lung. Urine excretion was observed. The PET imaging of the cardiovascular system in rats was clearly visualized with a low background ratio using ^18^F-VS-RBCs. As shown in Figure 3, ^18^F-VS-RBCs mainly stayed in the blood pool, suggesting good stability *in vivo*. At a late timepoint, urine activity was observed. As shown in Supplementary Figure 3, a small percentage of activity was released to the supernatant, which contains a hydrophilic motif. This may lead to the observed urine activity. Nonetheless, additional characterization would be done in a future study to further confirm it. Overall, ^18^F-VS-RBCs hold a great potential to imaging the cardiovascular system considering its slow clearance and stability profile.

**Table 1:** Biodistribution of ^18^F-VS-RBCs at 60 min after injection in normal rats (*n* = 5/group).

**Organ/tissue**	**Percentage of injected dose per gram of organ (%ID/g)**					
	**10 min**	**20 min**	**30 min**	**40 min**	**50 min**	**60 min**
Atrium	7.79 ± 0.17	8.28 ± 0.18	8.16 ± 0.22	7.94 ± 0.18	7.91 ± 0.17	7.72 ± 0.16
Spleen	3.05 ± 0.11	2.83 ± 0.16	2.98 ± 0.16	2.99 ± 0.17	2.87 ± 0.15	2.82 ± 0.11
Lung	1.03 ± 0.07	1.09 ± 0.08	1.18 ± 0.09	0.98 ± 0.09	0.95 ± 0.07	0.93 ± 0.06
Liver	1.75 ± 0.03	1.63 ± 0.03	1.53 ± 0.04	1.50 ± 0.04	1.39 ± 0.02	1.36 ± 0.02
Kidney	1.62 ± 0.04	1.50 ± 0.05	1.43 ± 0.02	1.37 ± 0.02	1.38 ± 0.02	1.29 ± 0.03
Bladder	0.92 ± 0.14	2.96 ± 0.16	3.17 ± 0.18	4.91 ± 0.19	5.02 ± 0.17	7.37 ± 0.17

#### Imaging Study of the Intra-Abdominal Hemorrhage Model on Rats

**Figure 4:**
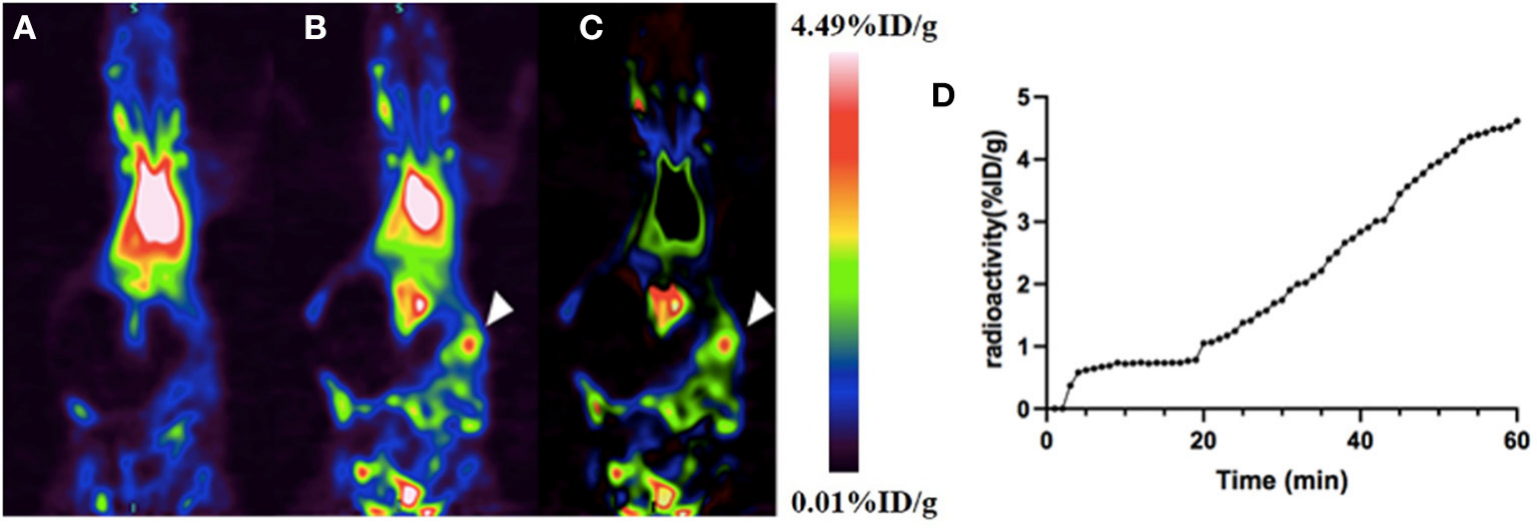
The application of ^18^F-VS-RBCs for PET imaging of the intra-abdominal hemorrhage model. The maximum-density projection image before **(A)** and after **(B)** bleeding and the subtraction image between the two **(C)**. The subtracted image showed heavy bleeding in the right abdomen (arrowheads). The time–activity curve of extravascular radioactivity in the abdominal hemorrhage area. Radioactivity continued to increase after manual puncture of the colon wall (18 min) **(D)**.

A dynamic PET scan was performed on rats to evaluate the feasibility of using this BPI agent for the diagnosis of intra-abdominal hemorrhage. Dynamic PET imaging of the rat gastrointestinal bleeding model showed that shortly after the colon wall puncture, a high aggregation site appeared in the abdomen, indicating the tracer had extravasated due to bleeding (Figures 4A–C). Time–activity curves showed that the agent increased steadily at the bleeding site: radioactivity was stable within 10 min and continued to increase thereafter (Figure 4D). ^18^F-VS-labeled RBCs successfully found the location and direction of abdominal bleeding over time. Although this study did not quantify the amount of bleeding, it can provide a rough estimation based on the blood radioactivity curve. Clearly, PET imaging of the rat intra-abdominal hemorrhage model demonstrated that ^18^F-VS-RBCs hold a great potential for applications in gastrointestinal bleeding. Furthermore, without dietary restrictions, the labeling procedure of ^18^F-VS-RBCs is simpler and shorter than the reported ^18^F-FDG-RBCs. We would also like to point out that the potential limitations of ^18^F-VS-RBCs include the requirement of clean space due to blood collection and *in vitro* labeling procedures. The radiation exposure toward operators could be high. Further improvements may focus on simplifying the labeling step by reducing the number of washing and streamlining the production process. Nonetheless, ^18^F-VS-RBC PET of normal animal and intra-abdominal hemorrhage model suggested that the agent could represent a new BPI agent.

## Conclusion

In this study, ^18^F-VS was synthesized and successfully labeled RBCs for BPI. The resulting ^18^F-VS-RBCs clearly visualized the cardiovascular system and extravascular blood in the abdominal hemorrhage model. Compared with existing cardiac blood pool agents, ^18^F-VS-RBCs could be prepared using a readily available precursor and simple procedure. The agent has prolonged retention time in cardiac blood pool with a high-quality image. The agent's uptake was not affected by blood sugar, which eliminated the need of fasting. Because ^18^F-VS-RBCs cannot penetrate the blood–brain barrier, it would be interesting to test its application in detecting bleeding in the brain in future studies.

## Data Availability Statement

The original contributions presented in the study are included in the article/Supplementary Material, further inquiries can be directed to the corresponding authors.

## Ethics Statement

The animal study was reviewed and approved by The Affiliated Hospital of Southwest Medical University.

## Author Contributions

XZ and LW contributed to the study design, the labeling process, the imaging scan, the data analysis and they contributed equally to this paper. XZ wrote the manuscript and LW revised the manuscript. WF, YF, CZ, TX, and JC were responsible for the integrity of the data and the accuracy of the data analysis. TZ and LZ contributed to precursor synthesis, the labeling and evaluation of the tracer stability during the revision of the work. The YC and ZL was responsible for revising for important intellectual content. All authors have read and approved the final manuscript.

## Conflict of Interest

The authors declare that the research was conducted in the absence of any commercial or financial relationships that could be construed as a potential conflict of interest.
