# Supplementary material for: 18F-PEG1-Vinyl Sulfone-Labeled Red Blood Cells as Positron Emission Tomography Agent to Image Intra-Abdominal Bleeding
Source: Front Med (Lausanne). 2021 Jul 5;8:646862. doi: 10.3389/fmed.2021.646862 (PMC8287037; doi:10.3389/fmed.2021.646862)
Supplement: Supplementary file 1 [file Data_Sheet_1.docx]

**Supporting Information**

^18^F-PEG1-Vinyl Sulfone-labeled Red Blood Cells as Positron Emission Tomography Agent to Image Intra-abdominal Bleeding

Xinyi Zhang^1,2,3,†^, Li Wang^1,2,3,†^,Wenhui Fu^1,2,3^, Yue Feng^1,2,3^, Chengrun Zeng^1,2,3^, Liu Zhou, ^1,2,3,4^, Tao Zhang^5^, Tingting Xu ^1,2,3^, Jianpeng Cao^1,2,3^, Zibo Li^5,*^, Yue Chen^1,2,3, *^

**Content**

GeneralInformation..............................................................................................................................1

Scheme.S1 Preparation of ^18^F-VS...................................................................................................1

Fig.S1 Purification of ^18^F-VS on a semi-preparative HPLC..................................................1

Fig.S2 Radio purity of the separated ^18^F-VS................................................................................2

Fig.S3 Micro-PET/CT images of normal mouse at different time point post injection with ^18^F-VS-RBCs.................................................................................................................................2

Fig.S4 HPLC spectra andradioactivity count chart of supernatant after ^18^F-VS-RBCs incubation for 30min and 120min..................................................................................................3

Fig.S5 RBCs incubated with different concentration of ^19^F-VS in saline….……….…3

**General Information:**

^18^F-labeled VSconjugate was analyzed by using radio HPLC. HPLC method: C-18 Column: TYPE AQ 5μm, SIZE 4.6mm × 250mm, col.No.A6AD 10292. Solvent A: ultrapure water mixed with 0.1% volume ratio of trifluoroacetic acid, solvent B: acetonitrile mixed with 0.1% volume ratio of trifluoroacetic acid. (0–2 min, 5% solvent B, isocratic; 2–22 min, 5%-95% solvent B gradient elution) was adapted to for separated [^18^F]-VS purity check. The column temperature is room temperature, the flow rate was maintained at 1 mL/min for elution, and the wavelength of the ultraviolet detector is 254 nm.

Scheme.S1 Preparation of ^18^F-VS


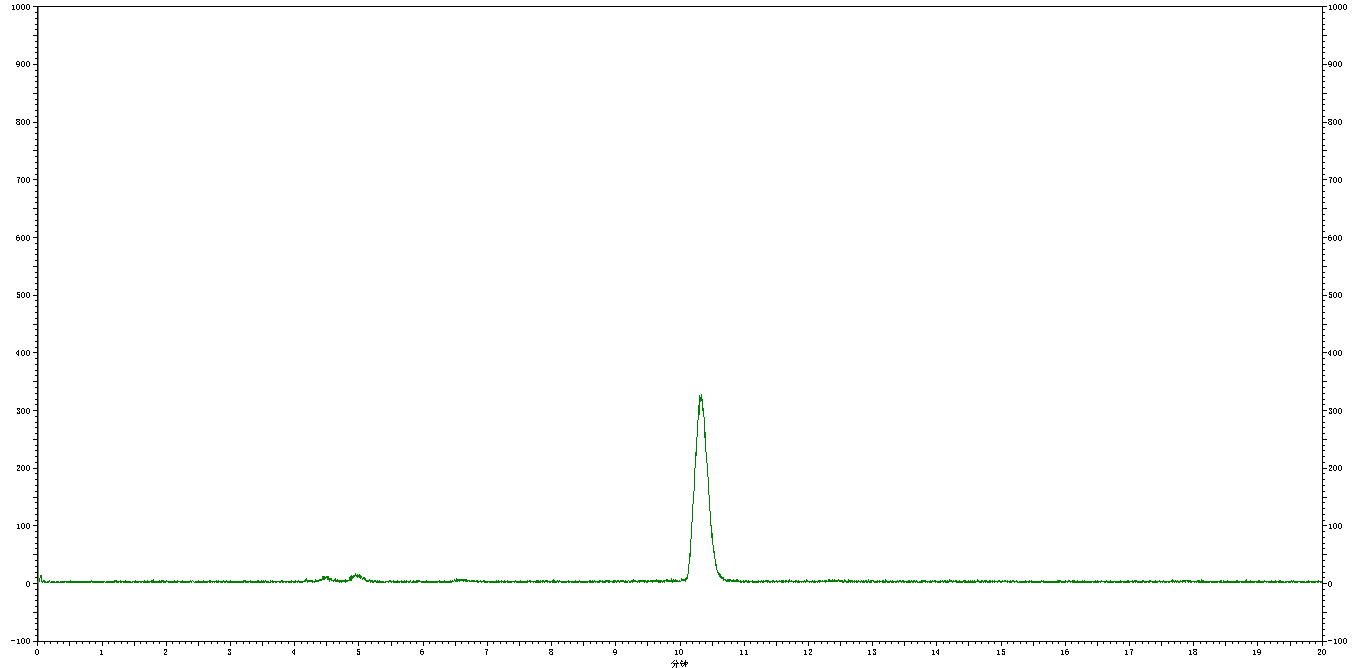


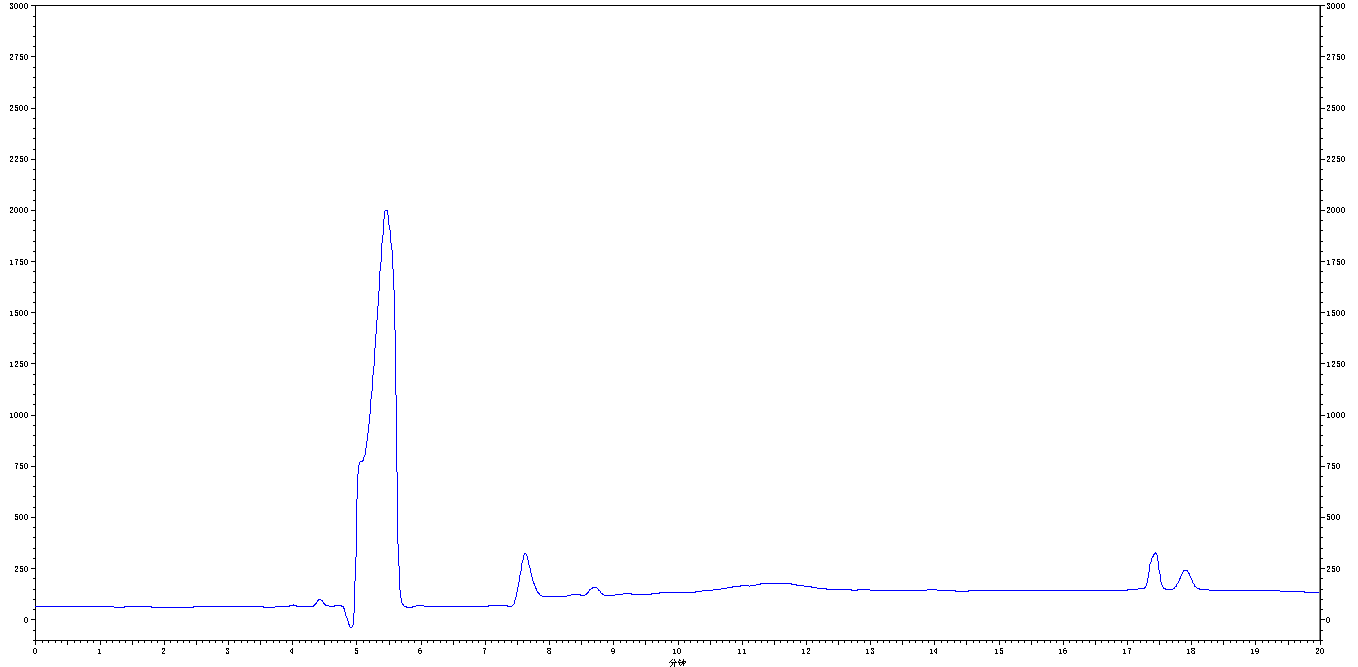


Fig.S1Purification of ^18^F-VSon a semi-preparative HPLC. The radioactivity trace (Above) andthe UV trace at 254 nm (Below) of the HPLC spectra.


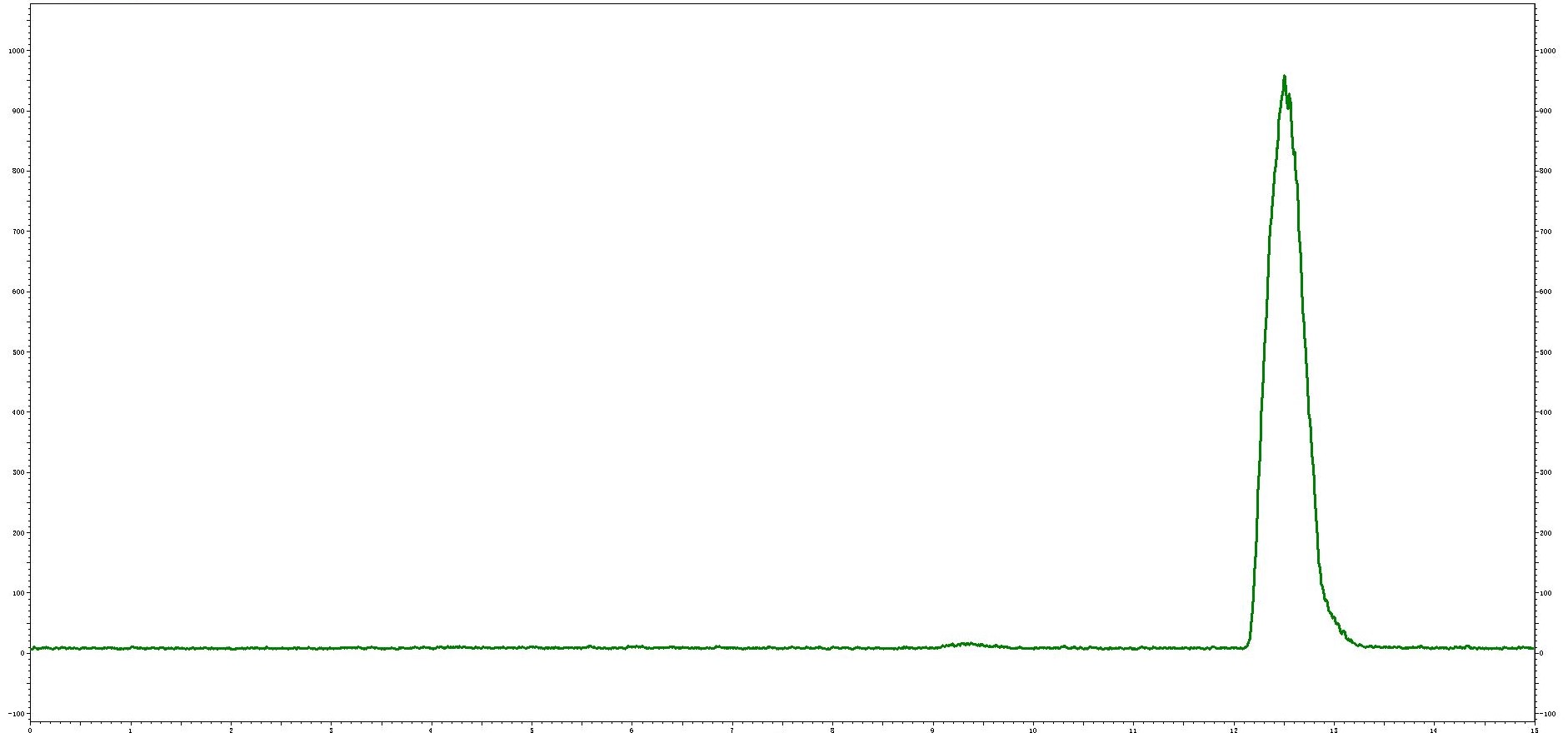

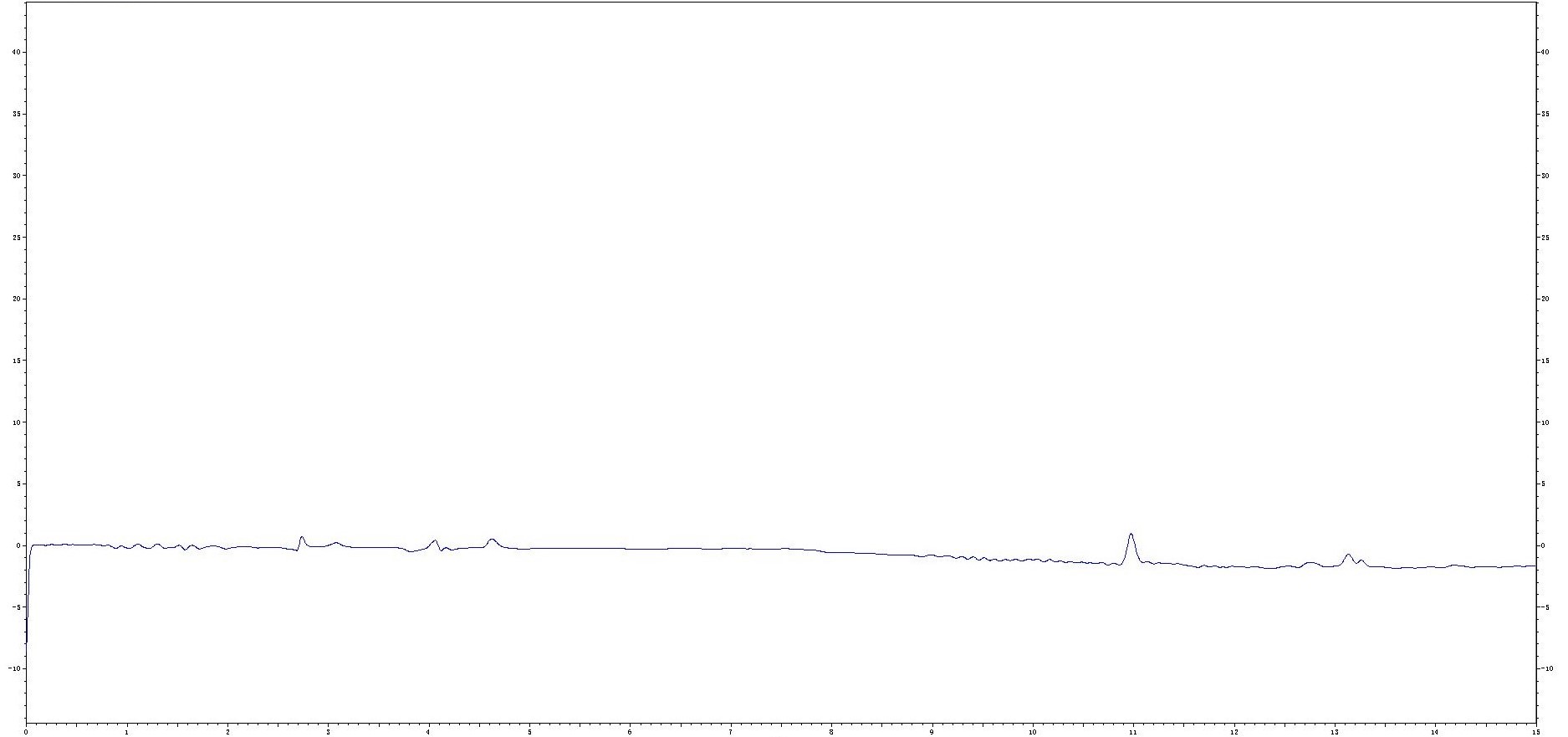


Fig.S2Radio purity of the separated ^18^F-VS. (Above) Radio trace of re-injection HPLC spectra for purified ^18^F-VS. (Below) UV trace of the re-injection HPLC spectra for purified ^18^F-VS at 254 nm.


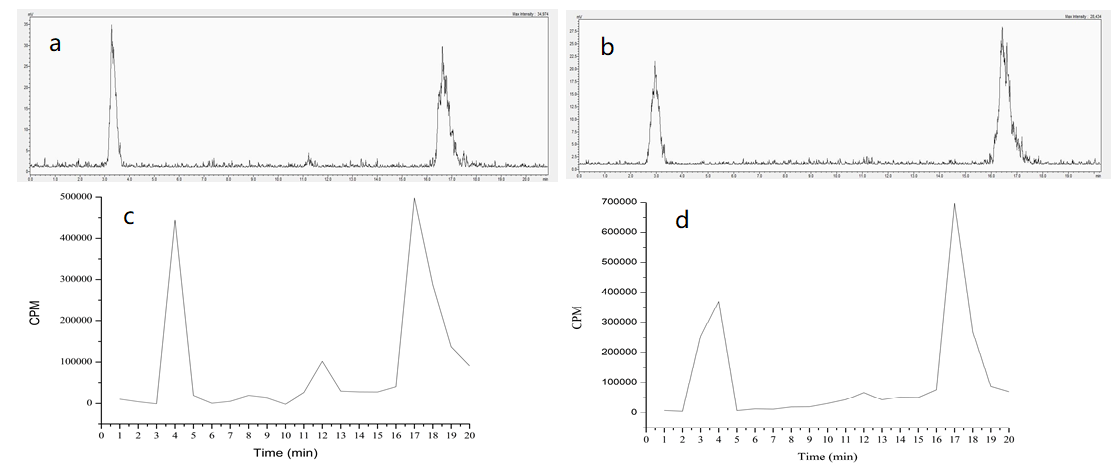


Fig.S3HPLC spectra of supernatant after ^18^F-VS-RBCs incubation for 30min and 120min (a:30min;b:120min);Radioactivity count chart of supernatant after ^18^F-VS-RBCs incubated for 30min and 120min (c: 30min; d: 120min) ; HPLC method (column: type, AQ 5 μm; size, 4.6 mm × 250 mm, col. no. A6AD 10292; solvent A: 0.1% trifluoroacetic acid water; solvent B: 0.1% trifluoroacetic acid acetonitrile; 0 to 2 min: isocratic elution of 15% solvent B; 2 to 20 min, 15−95% of solvent B; flow rate: 3 mL/min).


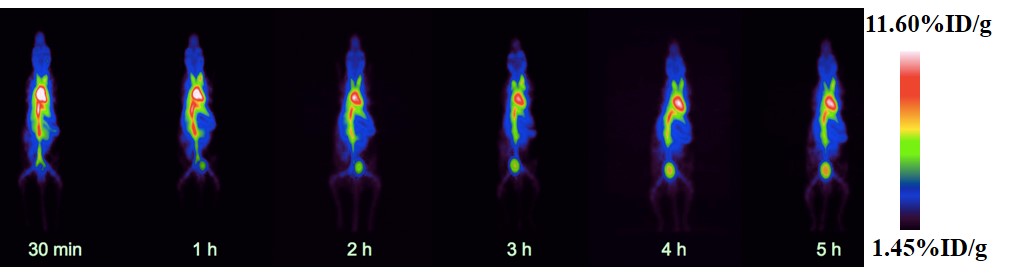


Fig.S4 Micro-PET/CT images of normal mouse at different time point post injection with ^18^F-VS-RBCs (3.7 MBq)


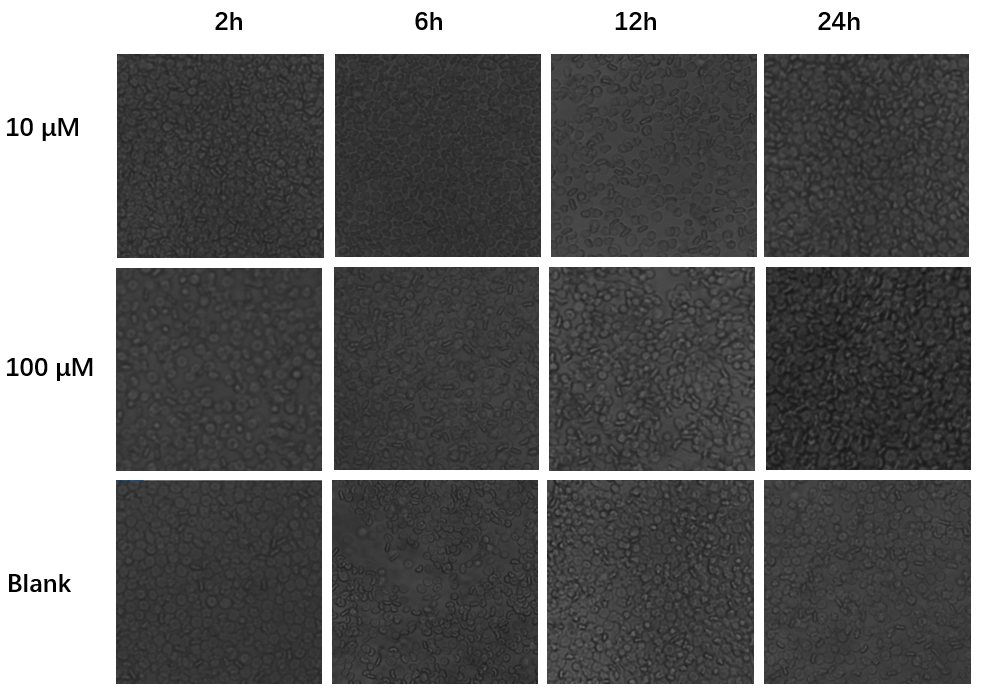


Fig.S5 RBCs incubated with different concentration of ^19^F-VS in saline
